# Economic Holobiont: Influence of Parasites, Microbiota and Chemosignals on Economic Behavior

**DOI:** 10.3389/fnbeh.2018.00077

**Published:** 2018-05-01

**Authors:** Petr Houdek

**Affiliations:** Faculty of Social and Economic Studies, Jan Evangelista Purkyně University in Ústí nad Labem, Ústí nad Labem, Czechia

**Keywords:** microbiota, chemosignaling, *Toxoplasma gondii*, parasites, risk preferences, time preferences, social preferences, economics

## Abstract

The article is a perspective on utilization of microorganisms and chemosignals in studying human economic behavior. Research in biological roots of economic development has already confirmed that parasitic pressure influenced the creation and development of cultural norms and institutions. However, other effects of microorganisms on human groups and individual decision-making and behavior are heavily understudied. The perspective discusses how parasitic infections, sexually transmitted organisms and microbiota (i.e., “human holobiont”) could causally influence risk-seeking behavior, impulsivity, social dominance, empathy, political views and gender differences. As a case study, the parasite *Toxoplasma gondii* and its influence on economic preferences, personal characteristics and human appearance are examined. I also briefly review how chemosignals influence decision-making, particularly in the social preferences domain. I mention some predictions that arise from the paradigm of economic holobiont for the economic science. The conclusion summarizes limitations of the discussed findings and the stated speculations.

## Introduction

No man is an island, entire of itself. His/her life is shaped by microorganisms (archaea, viruses, bacteria, protozoa) which help him/her digest food, synthesize important vitamins and fight against harmful microorganisms. Some microorganisms have developed the ability to affect our nervous and hormonal systems (Adamo, 2013; Mayer et al., 2014; Kramer and Bressan, 2015; Stilling et al., 2016; Allen et al., 2017).

Knowledge of the influence of microorganisms on specific long-term changes of human behavior, or personality dates back at least to the first descriptions of rabies. The infected individuals experience hydrophobia, which allows the virus to stay longer in their saliva, because they don’t drink, and they swallow less. Infected subjects then begin exhibiting aggressive behavior, which contributes to the transmission of the virus through bites (Baer, 1991). Impaired serotonin neurotransmission may account for the aggressive behavior (Jackson, 2016). The aim of this article is to show that plenty of other mind-altering microorganisms exist which, by more diminutive manipulations of the nervous and hormonal system, influence human behavior in areas that are of the interest of economics. For an overview, see Table 1.

**Table 1:** Examples of the influence of parasites, microbiota and chemosignals on economic decision-making.

Classes of deviances from the standard economicmodel (DellaVigna, 2009)		Examples of possible influence of parasites, microbiota and chemosignals on the behavior and decision-making
Nonstandard preferences	Time preferences (self-control problems)	Higher impulsive sensation-seeking among *Toxoplasma*-positive younger men (Cook et al., 2015)
		Lower diversity in gut microbiome could be associated with more impulsive behavior (Alcock et al., 2014)
	Risk preferences	*Toxoplasma*-positive subjects have lower novelty-seeking scores (Skallová et al., 2005)
		Exposure to anxiety odor/chemosignal increases risk taking behavior (Haegler et al., 2010)
	Social preferences	*Toxoplasma*-positive men are less cooperative, *Toxoplasma*-positive women are more cooperative (Lindová et al., 2010)
		Exposure to anxiety odor/chemosignal recruits empathy-related brain areas (Prehn-Kristensen et al., 2009)
		Probiotic formulation reduces aggressive thoughts (Steenbergen et al., 2015)
Nonstandard beliefs	Overconfidence or anxiety	*Toxoplasma*-positive subjects have higher extraversion and lower conscientiousness (Lindová et al., 2012)
		Odor/chemosignals of aggression can induce an anxiety reaction (Mutic et al., 2016)
Nonstandard decision-making	Limited attention	*Toxoplasma gondii* weakens long-term ability to concentrate (Havlíček et al., 2001)
		Exposure to fear odor/chemosignal enhances vigilance in women (Chen et al., 2006)
	Emotions, moods	Prevalence of latent toxoplasmosis explains neuroticism, uncertainty avoidance, and masculinity at national levels (Lafferty, 2006)
		Odor/chemosignals induce fear, disgust and happiness contagion in women (de Groot et al., 2012, 2015)
		Probiotic formulations alleviate psychological distress (Messaoudi et al., 2011)
		Administration of a bacteria (*Mycobacterium vaccae*) may build resilience and improve emotional responses under stress (Siebler et al., 2018)
	Social pressure	National parasite stress could strengthen traditionalism (Tybur et al., 2016)
		*Toxoplasma*-positive men have lower superego strength and protension (Flegr and Hrdý, 1994)

## Impact of Microorganisms on Human Cultures, Values and Individual Behavior

It is impossible to overrate the influence of parasites if they could be the driving forces of evolution in general (Decaestecker et al., 2007) as well as the evolution of sex (Hamilton et al., 1990). Microbes also possibly shaped the high level of human cooperation and socialization. The explanation stems from the fact that selective pressure favors those microbes that achieve higher interaction among their hosts—horizontal transmission is then the most effective (Lewin-Epstein et al., 2017). Their impact on human cultures, institutions, and individual decision-making should thus be applied more broadly in economics (even if we omit the direct impact of diseases on human capital, saving, worker productivity, or medical costs; Sachs and Malaney, 2002; Eppig et al., 2010, 2011).

There is an already existing literature about the influence of parasite and infection stress on the development of cultural norms and values of individuals, respectively (Fincher and Thornhill, 2012; Spolaore and Wacziarg, 2013). High prevalence of infections correlates with conformity, preservation of cultural norms, and xenophobia; put simply, cultural norms operate as prescriptions to avoid illness (Schaller et al., 2015). Consequently, there are lower mean levels of socio-sexuality, extraversion, and openness in regions with higher levels of infectious diseases (Schaller and Murray, 2008). Parasite stress strengthens family ties and religiosity, because in-group assortative sociality makes it easier to avoid infection from novel parasites (Fincher and Thornhill, 2012). Relatedly, national parasite stress strengthens traditionalism, i.e., conservative adherence to group norms (Tybur et al., 2016). However, the conclusions of the pathogen stress theory are controversial, since different authors suggest distinct interpretations (Hackman and Hruschka, 2013; Hruschka and Henrich, 2013; Hruschka and Hackman, 2014); also see “Sexually Transmitted Diseases and Beauty Premium, Impulsivity and Personality Disorders” section.

The prevalence of toxoplasmosis (see “*Toxoplasma gondii*” section) seems to be associated with neuroticism, uncertainty avoidance and masculinity at national levels (Lafferty, 2006). In development economics, Maseland used the national prevalence of *Toxoplasma gondii* as an instrument for cultural variation. “Increased vigilance and reduced moral consciousness make [*Toxoplasma*-infected] people more opportunistic and suspicious of the behavior of others, reducing cultural trust levels in society… lower self-confidence makes people less willing to stand up to authorities and pessimistic about the success of social interactions.” (Maseland, 2013). The study suggests that cultural effects of higher prevalence of *T. gondii* could be a strong predictor of institutional quality and long-term economic performance.

### Toxoplasma gondii

Most studies about the influence of parasites on individual human decision-making concern toxoplasmosis, caused by the protozoan parasite *T. gondii* (Webster, 2001; Flegr, 2013b, 2017; Houdek, 2017a,b). *T. gondii* infects about one third to one half of the human population and, unlike many other infectious diseases, it is widely spread in developed countries; more than 1 million new infections are estimated to occur each year in the United States (Jones and Holland, 2010). In immune-competent humans, infection by *T. gondii* is asymptomatic or manifest with mild flu-like symptoms (Weiss and Dubey, 2009).

*T. gondii* gained the ability to manipulate its host (typically mice, however potentially any warm-blooded animal) and to transmit to its definitive host (any feline species) by predation. The manipulative abilities of *T. gondii* manifest in both direct effects—infected mice have slower reactions (Webster, 2007), and they exhibit complex changes in behavior—infected mice lose fear of cats and cat urine starts attracting them (Berdoy et al., 2000; Flegr and Markoš, 2014). The manipulation of mice results in the parasite getting a higher chance of entering its final host, where it can sexually reproduce. The same effects are observed in humans—their reactions are slower (Havlíček et al., 2001) and attractiveness of cat odor increased for infected men but decreased for infected women (Flegr et al., 2011).

*T. gondii* is able to manipulate the neuroendocrine system, affects metabolism of dopamine (Prandovszky et al., 2011) and probably testosterone (Lim et al., 2013) and serotonin (Flegr, 2013a). The studies also identified correlation between toxoplasmosis and several mental illnesses (whose occurrence is connected with the aforementioned hormones and neurotransmitters): schizophrenia (Torrey et al., 2012), autism (Prandota, 2010), mood disorders (Pearce et al., 2012) and suicidal inclination (Ling et al., 2011); but see Sugden et al. (2016).

*Toxoplasma*-positive people (i.e., *Toxoplasma*-seropositive individuals) have an increased risk of traffic and work accidents (Flegr et al., 2002; Alvarado-Esquivel et al., 2012), and various impairments of cognitive functions (Gale et al., 2015). Infected individuals manifest a decreased tendency for novelty seeking, decreased conscientiousness and higher extraversion (Flegr et al., 2003; Skallová et al., 2005; Lindová et al., 2012). Some personality changes are in the opposite direction in men and women (Flegr, 2010). For instance, *Toxoplasma*-positive men had lower superego strength and protension (Flegr and Hrdý, 1994), whereas *Toxoplasma*-positive women score higher in affectothymia, alaxia and untroubled adequacy and self-sufficiency (Flegr et al., 1996). Nevertheless, toxoplasmosis is also associated with higher aggression among women (but not among men) and with higher impulsive sensation-seeking among younger men (Cook et al., 2015). On the other hand, our research shows that infected people are more patient, and the association is mediated by the Rh blood group (Houdek et al., 2017); while RhD heterozygotes are protected against negative effects caused by *T. gondii* infection (Novotná et al., 2008). In general, the intensity of parasite manipulations of the hosts (i.e., their cognitive impairments, personality changes) amplifies with the length of the infection, implying possible causal influence of the parasite (Flegr, 2013b).

As far as I know, only a few laboratory experiments with real incentives observed the influence of *T. gondii* on economic decision-making. In the Dictator Game, infected men were less generous, and in the Trust Game, women were more trusting, however, the effects were small or insignificant (Lindová et al., 2010). An insignificant influence of toxoplasmosis on risk attitude and loss aversion in a small sample of women is reported by Lanchava et al. (2015). If the effects of toxoplasmosis are real, there is an untapped opportunity to use it as a variable in studies about the impact of cognitive skills, personality traits, or non-cognitive skills on labor market outcomes, job (self-)selection, entrepreneurship, well-being, et cetera (Bowles et al., 2001; Knudsen et al., 2006; Borghans et al., 2008; Chiteji, 2010; Cubel et al., 2016).

In some cases, toxoplasmosis demonstrates a different effect on men and women. The reasons for these differences remain unknown insofar; however, they could be different physiological mechanisms responsible for the stress response (Lindová et al., 2006). It is possible to use *T. gondii* in research on gender differences (Croson and Gneezy, 2009)—e.g., to see whether a higher gender pay gap exists in areas, sectors and companies with a higher prevalence of toxoplasmosis.

Moreover, there are many more microbes that affect humans similarly, e.g., chlorovirus is associated with a decrease in cognitive functioning (Yolken et al., 2014), cytomegalovirus (β-herpesvirus) could cause depression, anxiety and increase overall psychological morbidity (Phillips et al., 2008). Other infectious agents could have similar impacts (Wang et al., 2014). On the other hand, research (insofar on mice) shows that some infections could improve stress-related emotional behavior (Lowry et al., 2007) and even induce antidepressant-like responses (Siebler et al., 2018).

### Sexually Transmitted Diseases and Beauty Premium, Impulsivity and Personality Disorders

In theory, hosts may express more adaptive behavior and traits when infected by parasites (Weinersmith and Earley, 2016). The logic of evolution commands that sexually transmitted parasites, bacteria and viruses could enhance the sexual attractiveness of their hosts, because that way they achieve a higher chance of transmission to another host.

Sporadic evidence exists that *T. gondii* (which can be in some cases sexually transmitted; Flegr et al., 2014) enhances sexual attractiveness of infected brown rats males (Hari Dass and Vyas, 2014). Although the mechanism of achieving higher sexual attractiveness in the infected individuals is yet to be discovered, there are independent findings that *T. gondii* increases the level of testosterone in male rats (Lim et al., 2013). Men infected by *T. gondii* have higher body height, and are perceived as more dominant and masculine by women, a trait positively correlated with testosterone levels (Flegr et al., 2005; Hodková et al., 2007). These findings support the possibility that the higher level of testosterone in infected men could be the consequence of the infection and, ultimately, the result of the parasite’s host manipulation to transmit easily by sexual intercourse. Since testosterone relates to risk-seeking behavior, social dominance, lower empathy, reduced cognitive reflection, and so on (Apicella et al., 2008; Coates and Herbert, 2008; Rilling and Sanfey, 2011; Nave et al., 2017), a speculation arises whether *Toxoplasma*-positive subjects manifest these traits more intensely.

As humans can be infected by many bacterial and viral Sexually Transmitted Diseases (STDs), there is an unexploited opportunity to examine whether some of the microorganisms enhance the (sexual) attractiveness of their host (Heil, 2016) and provide another advantage for him. The potential parasite influence on attractiveness could impact many economic variables, in which influence of attractiveness was observed (Hamermesh and Biddle, 1994; Langlois et al., 2000) gaining a job position (Ruffle and Shtudiner, 2015), higher earnings (Scholz and Sicinski, 2015), probability of election to a political seat (Berggren et al., 2010) or the size of debt interest rate (Ravina, 2012); but see Kanazawa and Still (2017).

Anything that makes hosts have promiscuous sex more often will be beneficial to STDs. It could be speculated that STDs could *cause* positive attitudes toward sex, lower xenophobia and conservatism, increased dominance in men, increased submissiveness in women; higher impulsivity and creativity, lower conscientiousness and more personality disorders to disrupt long-term relationships in favor of short-term ones, et cetera (Miller and Fleischman, 2016).

## Microbiota, Gut–Brain Axis

It is surprising that the impact of food on economic decision-making is not widely studied. So far, it was reported that, for instance, eating spicy food increases risk taking (Wang et al., 2016), bitter taste was found to promote hostility (Sagioglou and Greitemeyer, 2014), sweet taste should increase agreeableness and helping behavior (Meier et al., 2012), etc. However, apart from some sort of priming, the studies do not present any theory that would physiologically explain why the phenomena occur. The aim of this section is to suggest an inspirational association between microbiome and human behavior.

Our intestinal tract is packed with up to 40 trillion microbes (Sender et al., 2016), and because we are born germ-free, the microbes have to come from the outside world (Ley et al., 2006). During and after birth, the newborn is populated with indigenous microbes (Dominguez-Bello et al., 2010). The microbiota (microbes living inside us and on us) then systematically affect development and function of organs, including the brain (the gut-brain axis is a bidirectional communication channel via neural, endocrine, and immune pathways; Mayer et al., 2014). Although gut microbiota doesn’t change much during adulthood, there are significant differences in its composition among people as well as cultures (Wu et al., 2011). The impact of geography and culture on alimentation is obvious, nevertheless, there is no study that examines the impact of microbiota on human culture.

As demonstrated by Lozupone et al. (2012): “Classification of people as lean or obese can be made solely on the basis of their gut microbiota with 90% accuracy,” and the causal effect can be discussed: “… mice that were raised germ-free and then colonized with the microbiota from an obese mouse gained fat more rapidly than those colonized with the microbiota of a lean mouse”. The absence of the right bacteria leads to an abnormal organ function (Heijtz et al., 2011). A study demonstrated that germ-free mice manifest abnormal physiological reactions to stress. This state is however reversible by probiotic-induced recolonization with bacteria *Bifidobacterium infantis* (Sudo et al., 2004). Microbiota produce neurochemicals which are analogical to the mammalian ones and which impact mood and behavior; e.g., 50% of dopamine and the majority of serotonin have an intestinal source. It was also proven that microbes change the receptor expression and a range of neural mechanisms (for reviews, see Alcock et al., 2014; Mayer et al., 2014). Gut microbiota profiles are associated with differences in emotional and attentional processing (Tillisch et al., 2017) and with deficits in normal social behavior (Sherwin et al., 2017). A study (Desbonnet et al., 2014) has found significant social impairments in germ-free mice, particularly in males.

Microbiota are possible to manipulate by probiotics, antibiotics, or dietary interventions offering an easy approach to study their influence on economic preferences and behavior; for example in cooperation (Rabin, 1993), trust (Fehr, 2009) and deception (Gneezy, 2005) games. The exact impact of microbiota on human decision-making is a matter of research in progress, however, an implication from the few pilot studies can be drawn (Kramer and Bressan, 2015; Sandhu et al., 2017)—as in case of mice, microbiota can have an impact on mood, cognitive functions and processing of social signals. It was found that e.g., probiotic formulations alleviate psychological distress (Messaoudi et al., 2011) and reduce cognitive reactivity to sad mood by reducing aggressive thoughts (Steenbergen et al., 2015).

## Chemosignals

The parasite *Plasmodium falciparum*, which causes malaria, is able to make its human host more attractive for its vector, the mosquito *Anopheles gambiae*, which enhances the probability of being transmitted (Lacroix et al., 2005). The mechanism underlying this manipulation is yet unknown, but the parasites likely change the infected individual’s breath or body odor (or it’s intensity by fever). The effect of chemosignals, being odors produced by the human body, is not restricted only to the parasite’s manipulative efforts. Based on animal studies, Archie and Tung (2015) conclude: “[M]icrobes can also affect host’s social behavior. One of the main ways these effects arise is through chemical signals: considerable evidence indicates that some animals cultivate odor-producing bacteria in their scent glands… [There are] correlations between host traits (e.g., sex, dominance rank, social group membership), the bacterial communities living in scent glands, and the volatile compounds that emerge from these glands”.

In case of humans, Semin and de Groot (2013) state that neuronal processing of human body odor differs from non-endogenous odors due to skin-gland excretions and bacterial activity. Therefore, it is possible to discuss that human chemosignals are processed through neuronal relays responsible for the processing of social information (and not only within olfactory brain areas). That allows them to gain a stronger social dimension. Moreover, humans (along with e.g., dogs or rats) are extremely capable of odor detection, they are able to detect odorants at concentrations as low as three droplets in an Olympic-size swimming pool (Yeshurun and Sobel, 2010).

I do not know of any economic study that observes changes in behavior based on the odor of others. However, psychological studies have already shown that happiness (de Groot et al., 2015), fear, or disgust (Zhou and Chen, 2009; de Groot et al., 2012) can be transferred by the means of odors. Even sniffing negative-emotion-related odorless tears from women can reduce men’s sexual arousal, and salivary levels of testosterone (Gelstein et al., 2011). On the other side, a small study (Mutic et al., 2016) used body odor of men in a boxing session, in which they naturally behave aggressively with the goal to harm, to produce an anxiety-related stress response in participants. This relation is also applicable vice versa: anxiety chemosignals induce higher risk taking behavior (Haegler et al., 2010). Naturally, human chemosignals play a crucial role in human mate choice, sexuality and mother–infant bonding (Lübke and Pause, 2015).

de Groot et al. (2017) summarize the existing evidence: “[H]uman odorants become a medium by means of which information about dynamic states (e.g., emotions…) and enduring characteristics (e.g., age, gender, individuality, personality) can be transferred from a sender to a receiver.” The possible applicability of these findings in organization and labor economics or human resources management studies is clear. Furthermore, it could be used in marketing and sales. For instance, by measuring 100 trace gas species in a cinema, a study found out that specific film events, namely “suspense” or “comedy” caused viewers to change their emission of chemosignals (Williams et al., 2016). It concludes that the odors generated by the audience have a potential to alter the viewers’ rating of a movie.

## Conclusion

The article introduced several recommendations on how economic research of judgment and decision-making could use the knowledge of influence of parasites, microbiota and chemosignals on human behavior. The proposed speculations have many limitations. The reported studies are usually small-scale, underpowered, non-preregistered, and using a very specific sample of participants. They are based on questionnaires with hypothetical choices, they use multiple testing, selective reporting, etc. Therefore, there is a risk of false-positive results and overrating of the effects due to publication bias (Simmons et al., 2011; Asendorpf et al., 2013; Forstmeier et al., 2017). The predictions of economically relevant effects might not come true. It is crucial not only to replicate these findings, but also to do contextual replications and verify the robustness of the published effects.

Many empirical facts are not sufficiently established or examined as well; e.g., no specific human pheromone has been reliably identified (Wyatt, 2015). Some of the discovered effects contradict each other. Some effects may simply be the result of a prolonged illness. In the case of gut microbiome’s influence on the mind, Forsythe et al. (2016) conclude: “there are no clear indications that altering the resident microbiota would be of therapeutic benefit,” also a systematic review (Romijn and Rucklidge, 2015) didn’t find any evidence for probiotic efficacy in altering psychological outcomes.

## Author Contributions

The author confirms being the sole contributor of this work and approved it for publication.

## Conflict of Interest Statement

The author declares that the research was conducted in the absence of any commercial or financial relationships that could be construed as a potential conflict of interest. The reviewer SR and handling Editor declared their shared affiliation.
